# The Smell of Hypoxia: using an electronic nose at altitude and proof of concept of its role in the prediction and diagnosis of acute mountain sickness

**DOI:** 10.14814/phy2.13854

**Published:** 2018-09-05

**Authors:** Jonathan R. N. Lacey, Carlos Kidel, Jildou M. van der Kaaij, Paul Brinkman, Edward T. Gilbert‐Kawai, Michael P. W. Grocott, Michael G. Mythen, Daniel S. Martin, the Xtreme Everest 2 Research Group, S Abraham, T Adams, W Anseeuw, R Astin, B Basnyat, O Burdall, J Carroll, A Cobb, J Coppel, O Couppis, J Court, A Cumptsey, T Davies, S Dhillon, N Diamond, C Dougall, T Geliot, E Gilbert‐Kawai, G Gilbert‐Kawai, E Gnaiger, M Grocott, C Haldane, P Hennis, J Horscroft, D Howard, S Jack, B Jarvis, W Jenner, G Jones, J van der Kaaij, J Kenth, A Kotwica, R Kumar, J Lacey, V Laner, D Levett, D Martin, P Meale, K Mitchell, Z Mahomed, J Moonie, A Murray, M Mythen, P Mythen, K O'Brien, I Ruggles‐Brice, K Salmon, A heperdigian, T Smedley, B Symons, C Tomlinson, A Vercueil, L Wandrag, S Ward, A Wight, C Wilkinson, S Wythe, M Feelisch, E Gilbert‐Kawai, M Grocott, M Hanson, D Levett, D Martin, K Mitchell, H Montgomery, R Moon, A Murray, M Mythen, M Peters

**Affiliations:** ^1^ University College London Centre for Altitude Space and Extreme Environment (CASE) Medicine UCLH NIHR Biomedical Research Centre Institute of Sport and Exercise Health London United Kingdom; ^2^ Royal Free London NHS Foundation Trust London United Kingdom; ^3^ Respiratory Medicine AMC, University of Amsterdam Amsterdam Netherlands; ^4^ Anaesthesia and Critical Care Research Unit University Hospital Southampton NHS Foundation Trust Southampton United Kingdom; ^5^ Critical Care Research Area NIHR Respiratory Biomedical Research Unit University Hospital Southampton NHS Foundation Trust Southampton United Kingdom; ^6^ Integrative Physiology and Critical Illness Group Clinical and Experimental Sciences Faculty of Medicine University of Southampton Southampton United Kingdom

**Keywords:** Altitude sickness, breath tests, e‐Nose, hypoxia, volatile organic compounds

## Abstract

Electronic nose (e‐nose) devices may be used to identify volatile organic compounds (VOCs) in exhaled breath. VOCs generated via metabolic processes are candidate biomarkers of (patho)physiological pathways. We explored the feasibility of using an e‐nose to generate human “breathprints” at high altitude. Furthermore, we explored the hypothesis that pathophysiological processes involved in the development of acute mountain sickness (AMS) would manifest as altered VOC profiles. Breath analysis was performed on Sherpa and lowlander trekkers at high altitude (3500 m). The Lake Louise Scoring (LLS) system was used to diagnose AMS. Raw data were reduced by principal component (PC) analysis (PCA). Cross validated linear discriminant analysis (CV‐LDA) and receiver‐operating characteristic area under curve (ROC‐AUC) assessed discriminative function. Breathprints suitable for analysis were obtained from 58% (37/64) of samples. PCA showed significant differences between breathprints from participants with, and without, AMS; CV‐LDA showed correct classification of 83.8%, ROC‐AUC 0.86*;* PC 1 correlated with AMS severity. There were significant differences between breathprints of participants who remained AMS negative and those whom later developed AMS (CV‐LDA 68.8%, ROC‐AUC 0.76). PCA demonstrated discrimination between Sherpas and lowlanders (CV‐LDA 89.2%, ROC‐AUC 0.936). This study demonstrated the feasibility of breath analysis for VOCs using an e‐nose at high altitude. Furthermore, it provided proof‐of‐concept data supporting e‐nose utility as an objective tool in the prediction and diagnosis of AMS. E‐nose technology may have substantial utility both in altitude medicine and under other circumstances where (mal)adaptation to hypoxia may be important (e.g., critically ill patients).

## Introduction

The physiological responses to hypoxemia are diverse and the mechanisms that underpin human hypoxic adaptation remain unclear (Grocott and Montgomery 2008). Within critical care, there is substantial interindividual variation in patients’ response to hypoxemia and there is difficulty identifying those who will respond adversely (Grocott et al. 2007). Similarly, there is considerable variation in performance when individuals are exposed to hypobaric hypoxia at high altitude, and there is no reliable method to identify those at risk of developing acute mountain sickness (AMS) (Martin et al. 2010). These parallels have prompted research into healthy subjects at high altitude to provide novel insights into the (patho)physiology of hypoxic (mal)adaptation in critically ill patients (Grocott et al. 2007). Of particular interest are the Sherpa people who demonstrate extraordinary adaptation to high altitude yet show no conventional markers of improved systemic oxygen delivery (Gilbert‐Kawai et al. 2014). A better understanding of Sherpa physiology could provide candidates for improved management strategies in our sickest hypoxemic patients (Martin et al. 2013).

Volatile organic compounds (VOCs) are a diverse group of carbon‐based molecules, generated via various metabolic processes. Endogenous VOCs have a multi‐systemic origin and are transported within the blood before being excreted in exhaled breath. The number of VOCs in exhaled breath exceeds several thousand and the profile varies according to underlying inflammatory states(Buszewski et al. 2007; van de Kant et al., 2012). As such these compounds pose enormous potential as pathophysiological biomarkers. The Cyranose 320 (*Sensigent*, USA) is a handheld vapor analyser, known as an electronic nose (e‐nose). Although not designed for clinical use, it can be used to analyze exhaled VOCs. The 32 carbon polymer sensors absorb volatiles causing a change in the sensors’ electrical resistance. The magnitude and distribution of changes of resistance creates a specific pattern or “breathprint” for that sample. Analysis by pattern recognition algorithms can then discriminate between samples, without identifying individual molecular components. E‐noses have been shown to be capable of distinguishing between various respiratory diseases, including lung cancer and asthma (Machado et al. 2005; Dragonieri et al., 2007, 2009; Fens et al. 2009).

In this study, we hypothesized that the pathophysiology involved in the development of AMS would manifest as altered VOC profiles in exhaled breath. Furthermore, we hypothesized that those groups known to show superior adaptation to hypoxemia, namely Sherpa people, will express this physiological advantage in their breathprints. Specifically, breath analysis using an e‐nose could be used to identify individuals suffering from AMS and to distinguish resistant or susceptible individuals.

### Study objective

To develop a method for breath analysis, using an e‐nose (Cyranose 320) that is feasible to use in an austere high altitude environment. Secondary aims were to investigate if breath analysis during early exposure to high altitude hypobaric hypoxia can be used to: (1) diagnose those suffering from AMS; (2) identify those at risk of developing AMS; and (3) to distinguish Sherpas from lowlanders.

## Methods

### Ethical approval

Ethical approval was obtained from the Nepal Health Research Council (reference 139/2012) and the University College London Research Ethics Committee (reference 3750/006). Written informed consent was obtained from all participants and the study complied with the standards set by the Declaration of Helsinki.

### Participants

Participants were adult volunteer trekkers (aged over 18 years) recruited from two distinct population groups: lowland residents (primarily European with no Tibetan/Andean/Ethiopian ancestry, residing below 1300 m altitude) and indigenous Sherpas with confirmed Tibetan ancestry. Study eligibility was dependent upon good health (determined through physician review of a detailed health questionnaire).

### Study design

This study formed part of a research programme that made up the “Xtreme Everest 2” expedition to Nepal (2013) (Gilbert‐Kawai et al. 2015). Xtreme Everest 2 had a prospective observational design that has been detailed previously (Gilbert‐Kawai et al. 2015). The aim of the expedition was to investigate the physiological mechanisms involved in acclimatization and adaptation to hypobaric hypoxia. Participants were assigned into trekking groups which followed identical ascent profiles. Participants provided a single breath sample on reaching high altitude at Namche Bazaar (3500 m), before continuing on to Everest Base Camp (5300 m). Participants completed a daily diary describing any AMS symptoms at baseline and for the duration of the trek. The criterion for AMS diagnosis was a Lake Louise Score (LLS) of 3 or more, including the presence of a headache (Roach et al. 1993).

### Exhaled breath analysis

**Figure 1:**
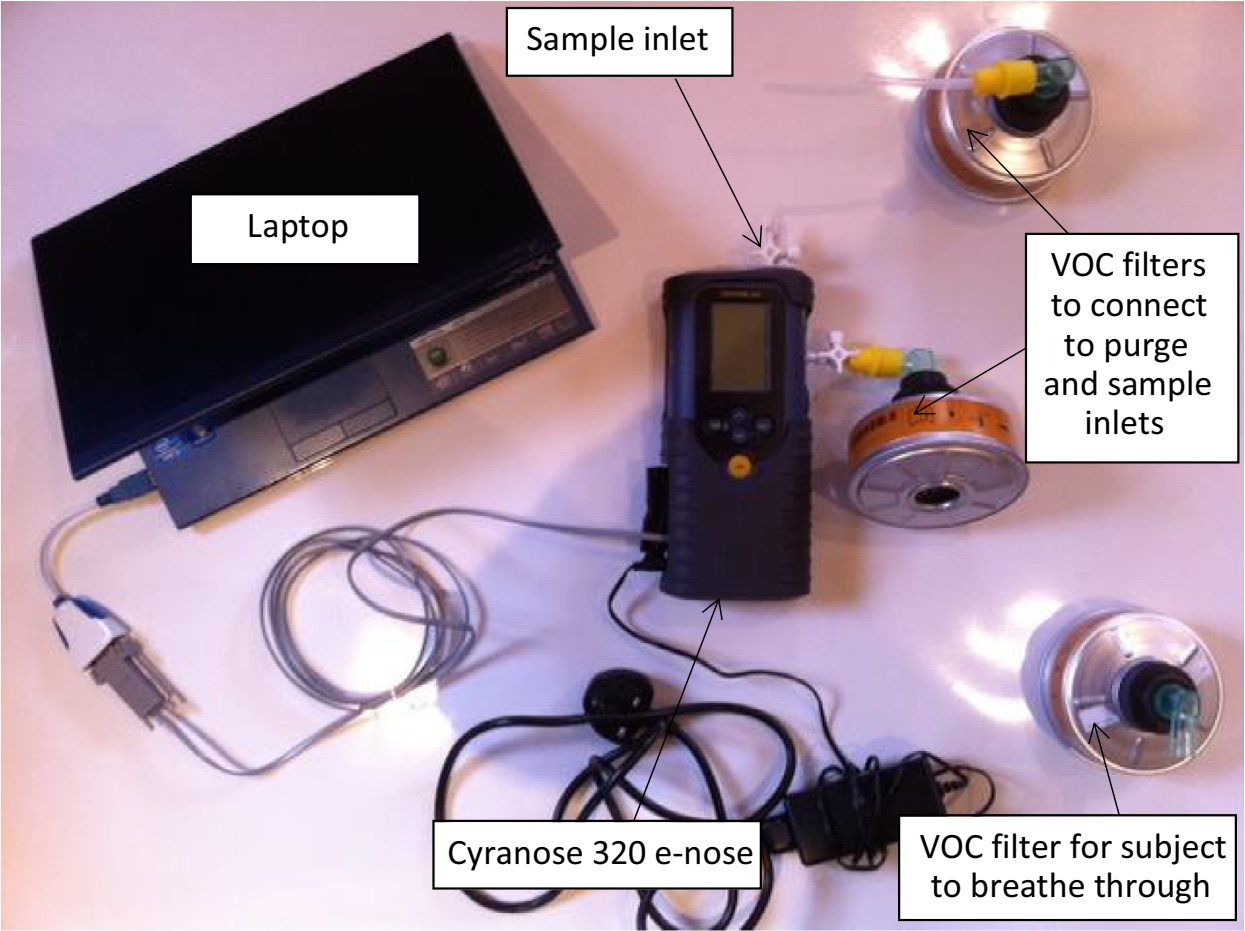
Photograph of e‐nose (Cyranose 320) and breath analysis apparatus.

A validated method for breath sample collection (Dragonieri et al. 2007) was modified to adapt to the logistical and environmental restrictions of the expedition. A major objective was to reduce exogenous contaminants. All subjects avoided eating/drinking, smoking, brushing teeth or using inhaler devices for 2 h prior to breath sampling. To minimize contamination by ambient VOCs, subjects underwent 5 min tidal breathing of VOC‐filtered air. This was achieved using a VOC filter (A2 vapor filter, *North Safety Products Europe*), to which an angle piece (elbow connector 15M‐22M/15F, *Intersurgical*, UK) was attached to allow subjects to inhale through the filter apparatus and exhale into ambient air. Nose clips were worn to prevent nasal entrainment of nonfiltered ambient air. An expiratory vital capacity breath was then collected into an inert Nalophan bag. This collection system was made by cutting 60 cm sections of double‐layered Nalophan sheet: one end was sealed using a plastic locking system (*Clip‐n‐Seal*, USA); the other end was sealed around a 20 cm section of polytetrafluoroethylene tubing using a cable tie. The polytetrafluoroethylene tube then served as a mouthpiece to allow subjects to provide their sample. After collection, the bag was immediately connected to the sample inlet of the e‐nose via the polytetrafluoroethylene tubing. VOC filtered air was used as reference air for the 60 sec baseline draw, followed by a 40‐sec sample draw and completed by 180 sec of purging with VOC filtered air (60 sec via the purge inlet and 120 sec via the sample inlet). The image in Figure 1 shows the e‐nose and apparatus set‐up.

### Data analysis

Offline analysis of raw e‐nose data was performed, using SPSS software (version 20.0). After verifying normal distribution, data were reduced by principal component analysis (PCA). PCA is a statistical procedure for reducing high‐dimensional datasets into smaller sets of linearly uncorrelated variables, called principal components, that retain the trends and patterns of the original data (Lever et al. 2017). After PCA, principal components with eigenvalues greater than 1 were retained for further analysis, which is in agreement with the Kaiser Criterion (Yeomans and Golder 1982). A bootstrapped‐independent samples *t*‐test was then used to assess if the preserved principal components were discriminative between groups. Based on the differentiating principal components, cross‐validated linear discriminant analysis (CV‐LDA) was performed and receiver operating characteristic (ROC) area under curve (ROC‐AUC, ±95% CI) was calculated to classify cases into categorical divisions. ANOVA and regression analysis were used to ascertain correlation between principal components and LLS for AMS.

## Results

Sixty‐four breath samples were obtained, from which 37 samples were suitable for analysis. Four samples were excluded from the first day of testing due to failed sample draws; changing the pump setting to high‐speed prevented this recurring. The remaining 23 exclusions were deemed anomalies and are discussed further under “study limitations”.

### Demographics and baseline characteristics

The 37 subjects were divided into 18 Sherpas and 19 lowlanders (median age 24.5 and 31.0 years, respectively), with similar male: female ratios. Four Sherpas and one lowlander were smokers. Two participants suffered from mild, well‐controlled asthma; there were no other respiratory diseases amongst the trekkers. There was a significantly greater proportion of lowlanders (72%) diagnosed with AMS (*P *<* *0.001). It was not possible to exclude AMS for one lowlander at Everest Base Camp because of missing diary data.

### Diagnostic ability of breath analysis with an e‐nose

**Figure 2:**
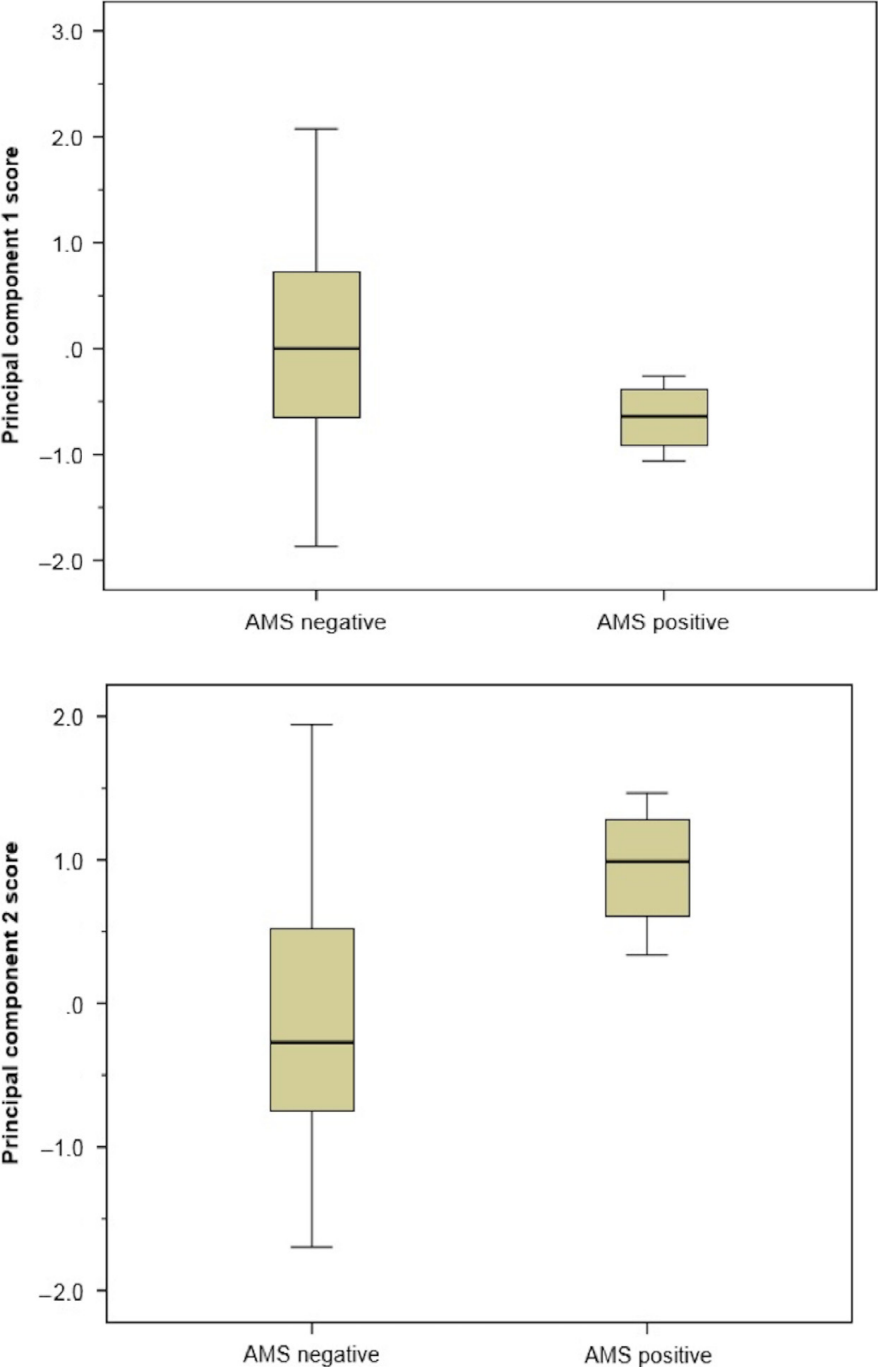
Boxplots comparing principal components 1 and 2 of AMS negative and AMS positive breathprints at Namche Bazaar.

**Figure 3:**
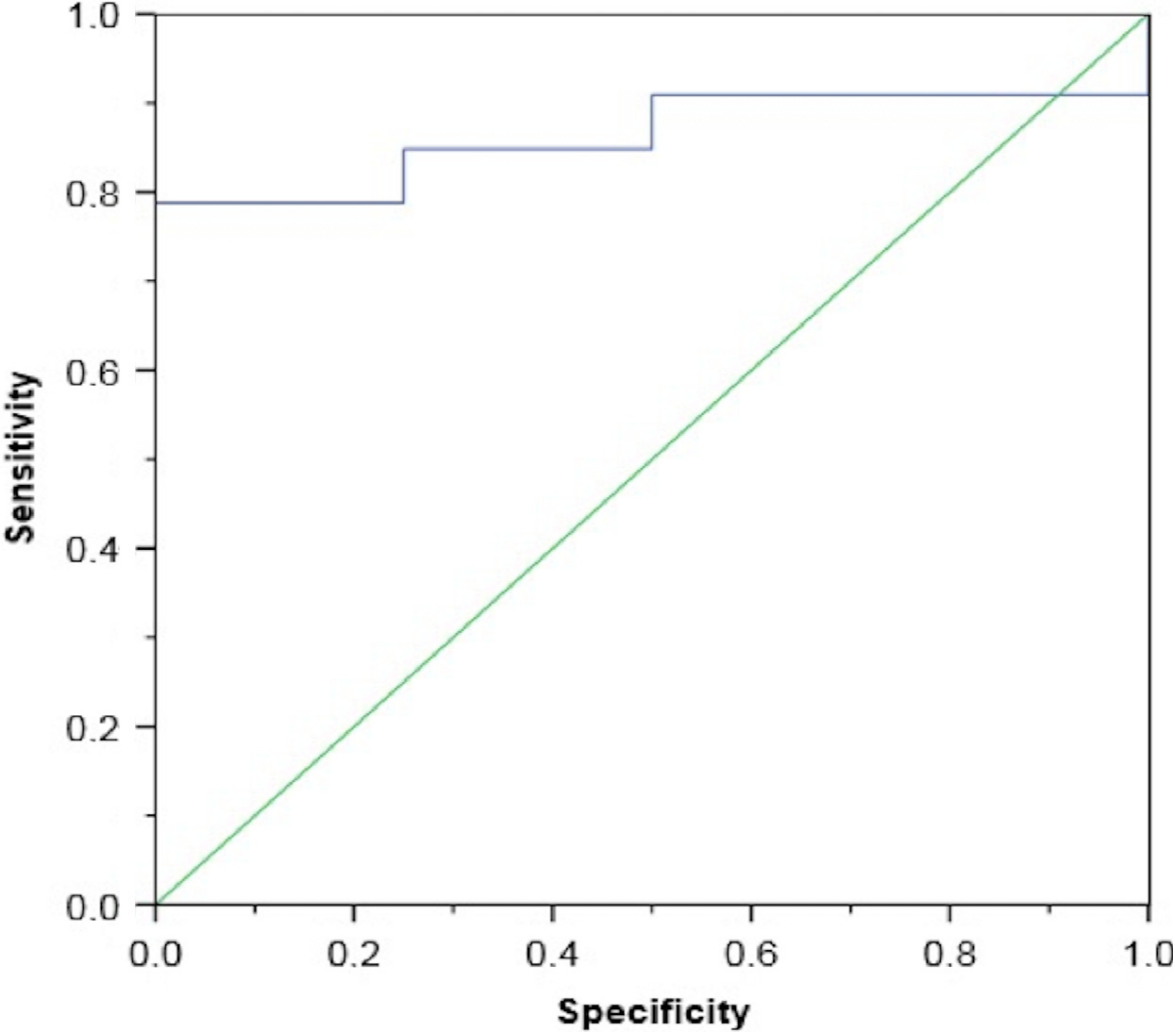
ROC Curve demonstrating discrimination between AMS positive and AMS negative breathprints at Namche Bazaar.

**Figure 4:**
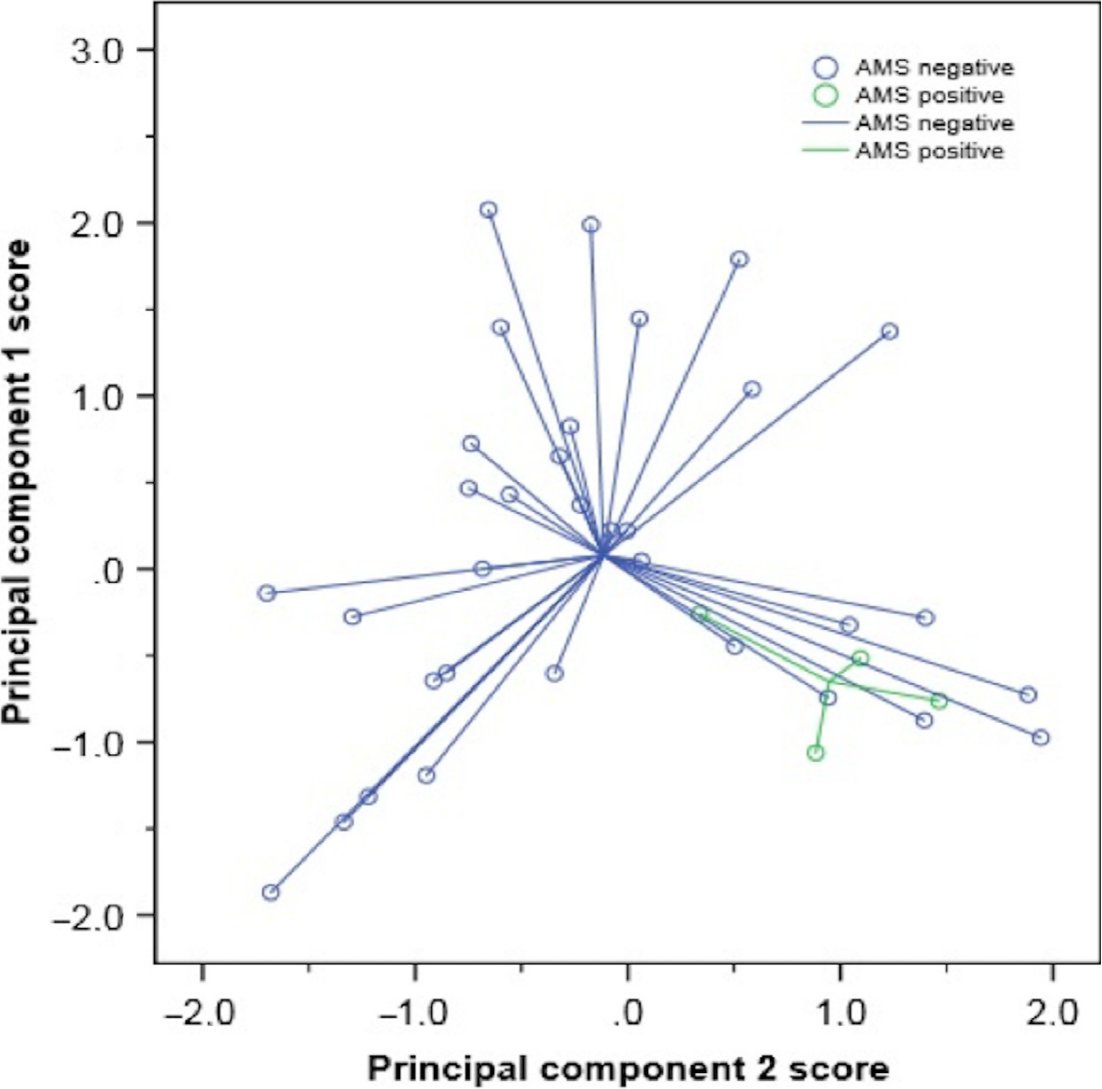
Scatterplot comparing AMS positive (green) with AMS negative (blue) breathprints at Namche Bazaar.

Four of 37 participants (all lowlanders) suffered from AMS at Namche Bazaar. From the original 37 breathprints produced by the e‐nose, three principal components with an eigenvalue larger than 1 were derived, capturing 83% of the variance within the total dataset. The subsequent bootstrapped independent samples *t*‐test between AMS positive and AMS negative participants resulted in significant outcomes for both principal component 1 and principal component 2 (*P *=* *0.006 and *P *=* *0.001, respectively) (Fig. 2). CV‐LDA showed correct classification of 83.8% of all cases and an ROC‐AUC of 0.86 ± 0.12 (Fig. 3). Sensitivity (0%) and specificity (94%) indicated that the model is unstable with the skew distribution of 33 AMS negative and 4 AMS positive participants. A plot of the two discriminative principal components illustrates the difference in mean values between AMS positive and negative, but shows no clear distinction between groups (Fig. 4).

### Correlation of AMS severity and breath analysis with an e‐nose

Maximum LLS for individuals was used as an indicator of severity of AMS. Missing diary data for one lowlander at Namche, although it did not affect AMS diagnosis, did prevent accurate calculation of total LLS and hence the subject was excluded from this subset analysis. ANOVA showed a significant difference between principal component 1 and maximum LLS at Namche Bazaar (*P *=* *0.02). Linear regression analysis indicates a correlation between both variables (*R* ^2^ = 0.22, *R* ^2^ adjusted = 0.20, *P *=* *0.004).

### Predictive ability of breath analysis with an e‐nose

The four participants diagnosed with AMS at Namche Bazaar were excluded. A fifth participant was excluded because of missing diary entries. Eleven out of the remaining 32 participants were diagnosed with AMS at some time in their trek from Namche to Everest Base Camp. Following PCA, pattern‐based breath analysis between participants who remained AMS negative and those that developed AMS showed a significant difference on principal component 1 (*P *=* *0.009). CV‐LDA showed correct classification of 68.8% of cases, ROC‐AUC 0.76 ± 0.18, with sensitivity of 46% and specificity of 81%.

### Ability of breath analysis with an e‐nose to distinguish between Sherpas and lowlanders

**Figure 5:**
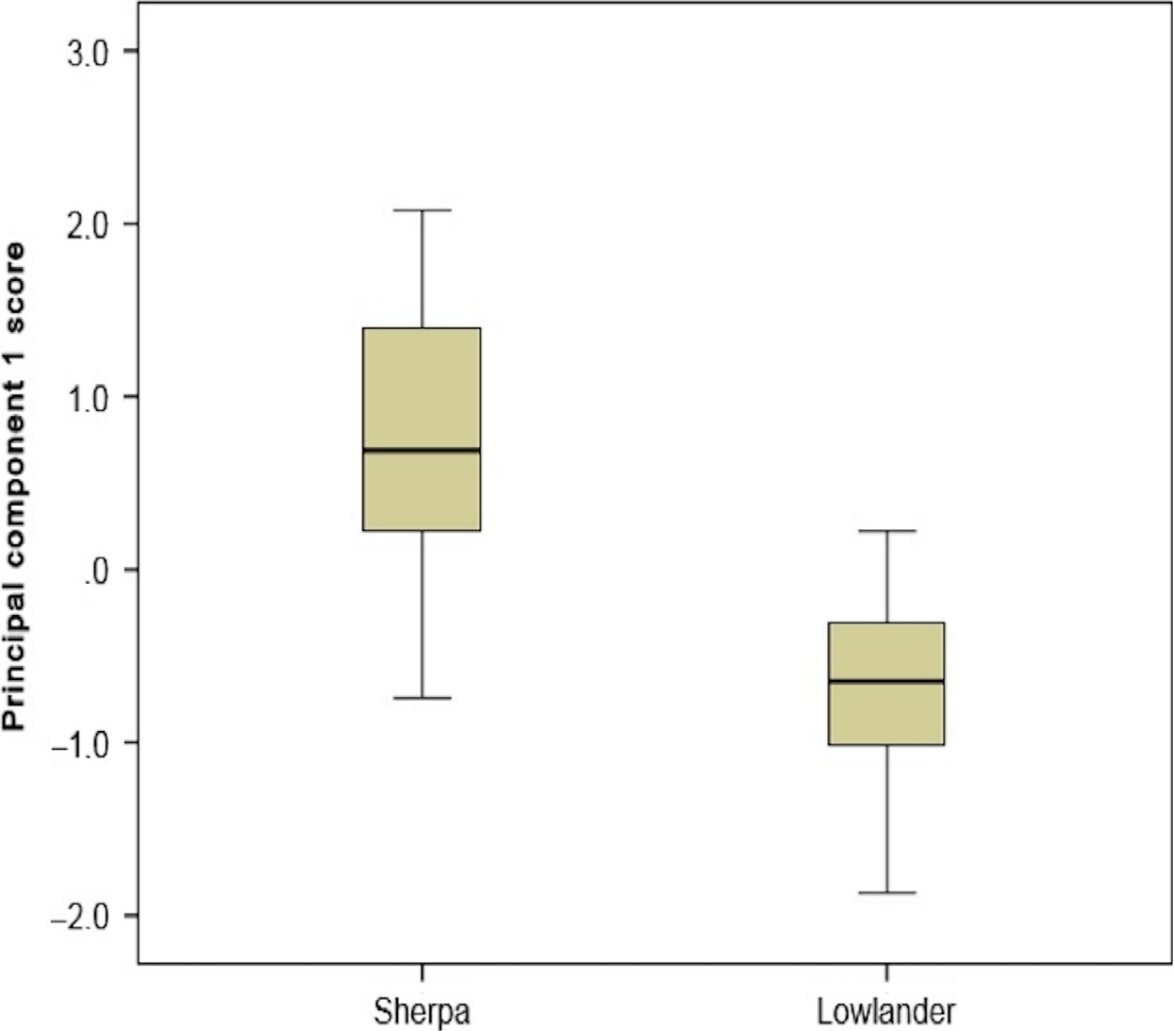
Boxplot comparing breathprints from Sherpas with Lowlanders.

**Figure 6:**
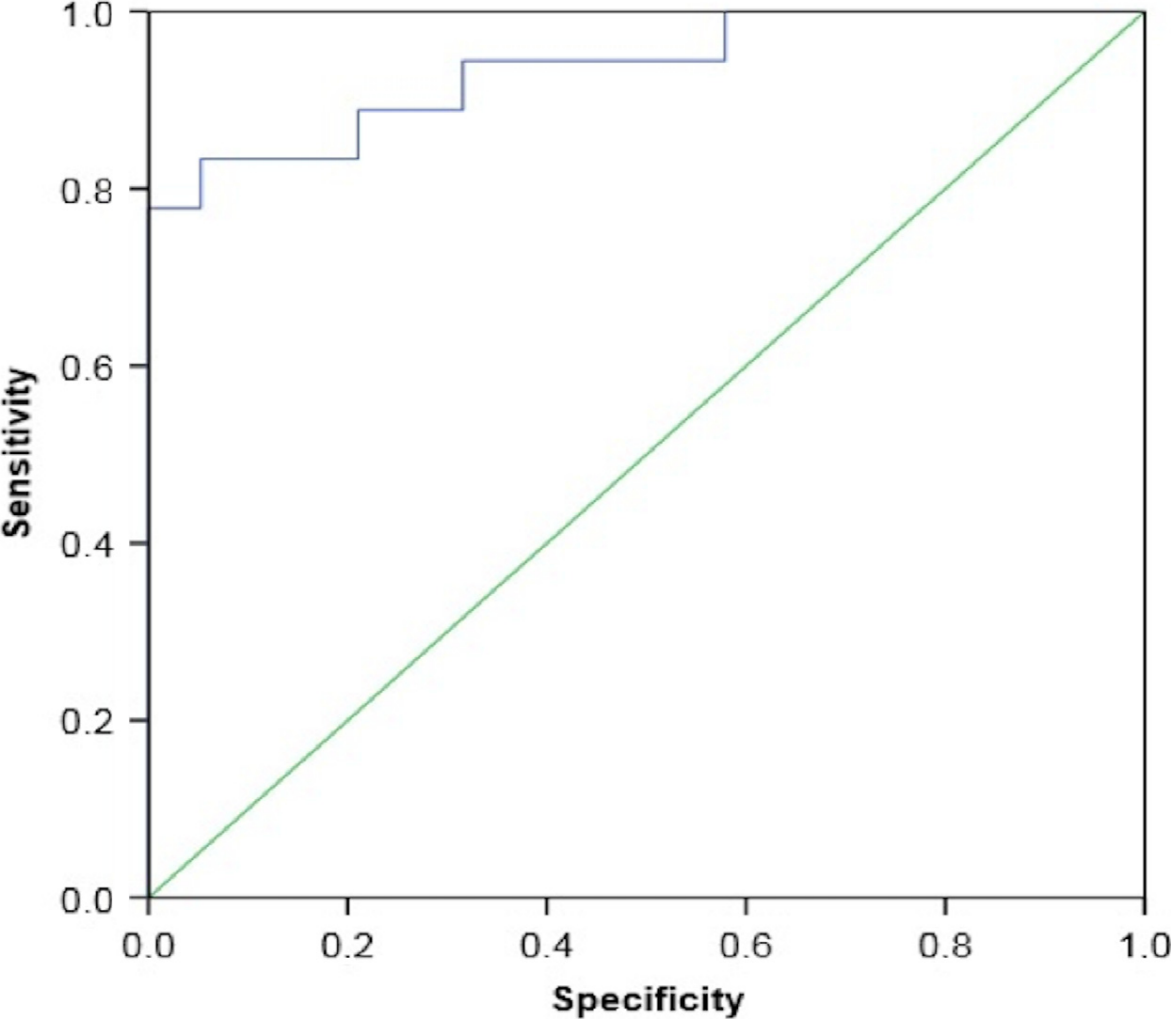
ROC Curve demonstrating discrimination between breathprints from Sherpas and Lowlanders.

PCA of Sherpas’ and lowlanders’ breathprints showed significant difference on principal component 1 (*P *=* *0.001) (Fig. 5). CV‐LDA showed correct classification of 89.2% of cases, ROC‐AUC 0.936 ± 0.08 (Fig. 6) with sensitivity of 94.7% and specificity of 83.3%.

## Discussion

This study demonstrated the first exhaled breath analyses, using the Cyranose 320 e‐nose at high altitude and it is the first to have investigated its role in the diagnosis and prediction of AMS. It is also the first time that an e‐nose has been used to compare breathprints from Sherpas with lowlanders, further developing the concept that different hypoxic response phenotypes may be reflected in the profile of exhaled volatiles.

### Diagnostic utility of breath analysis with an e‐nose

There is currently no objective method to diagnose and/or assess AMS. The results of this study provide a strong signal that breath analysis using an e‐nose can distinguish participants with AMS from those without. Significant differences between both principal components 1 and 2, indicate that VOC patterns within these two groups may be discriminatory. The low number of AMS positive participants, however, produced a skewed data set that makes it difficult to draw definitive conclusions about the diagnostic capability of e‐noses.

There are indications that breath analysis may have a role not only in AMS diagnosis but also in assessing severity. When trekkers were grouped according to their maximum LLS at the time of sampling, there was a significant difference between the respective sets of breathprints and linear regression showed a weak correlation between LLS and principal component 1.

In light of these results, it is worth considering whether the AMS negative plots that are grouped close to positive plots in Figure 4, do in fact represent volatiles generated from an evolving AMS pathophysiology. A recent study demonstrated a correlation between exhaled VOCs and the physiological responses to hypoxic environments (Figueroa et al. 2015).

### Predictive utility of breath analysis with an e‐nose

There was a significant difference between breathprints from trekkers who remained AMS negative compared to those who developed AMS later in the expedition; the e‐nose was able to identify those at risk of developing AMS with reasonable discrimination. There is currently no reliable method to risk‐stratify people for AMS. The ability to do so would provide a great advantage to medical teams, allowing targeted and timely interventions to prevent progression to AMS.

### Ability of breath analysis with an e‐nose to distinguish between Sherpas and lowlanders

There were provocative results for the ability of the e‐nose to distinguish Sherpas from lowlanders. This is an exciting outcome which supports the use of breath analysis to discriminate between people with different responses to hypoxia. It also suggests that analysis of VOCs in exhaled breath may help us to understand better the physiological mechanisms that result in (mal)adaptation to hypoxia. Alternatively, it is worth considering whether the differences observed in exhaled VOCs between Sherpas and lowlanders may be related to behavioral or genetic distinctions that are unrelated to hypoxia.

### Study limitations

Our design proved durable to the hostile conditions at high altitude, supporting the potential use of breath analysis as an objective assessment tool for AMS. However, the 27 exclusions detailed in the results merit further discussion about the reliability of the method under such conditions. The four failed sample draws may have been secondary to an incorrect pump speed for low ambient barometric pressure, since changing to a high‐speed setting resolved the issue. This is important for those wishing to use similar apparatus at altitude. The remaining 23 exclusions were unexplained anomalies: on four separate days, in which both Sherpas & lowlanders were tested, the raw sensor data was grossly abnormal for all trekkers and across all 32 sensors. The interindividual spread was similar but the absolute values were substantially different from samples analyzed on all other testing days. Including these results for data analysis would make interpretation unreliable. One plausible explanation may relate to variations in the ambient temperature and/or humidity; a problem that has been identified with e‐noses during fieldwork in the environmental sector (Capelli et al. 2014).

The availability of only one e‐nose and the logistical restrictions of the trek meant it was only possible to sample breath at one geographical location. The number of people diagnosed with AMS at the time of breath sampling in Namche Bazaar was low (four out of 37 subjects). This produced a skewed data set which made it difficult to comment with confidence on the e‐nose's diagnostic utility. A second reading at Everest Base Camp, where the proportion of AMS positive subjects was greater, would have helped draw clearer conclusions. Likewise, additional breath analysis at sea level would have helped distinguish if there were differing exhaled VOC profiles between Sherpas and lowlanders independent of the effects of altitude i.e., are the demonstrated differences in VOCs a result of a constitutive or inducible physiological process.

The equipment used for breath collection and sampling was not designed for the purpose and substantial efforts were made to minimize contamination from components and ambient air. However, specifically designed equipment which has undergone external validation would help substantiate results for this and future projects. Furthermore, we would welcome studies to clarify the impact of environmental variables (i.e., ambient temperature, humidity, barometric pressure) on e‐nose breath analysis and to support the development of standardized methodology for field‐based research.

### Possible mechanistic explanations

Evidence suggests there is an intimate relationship between hypoxia and inflammation that is primarily mediated by hypoxia inducible factor (Eltzschig and Carmeliet 2011). Nitric oxide production, a hallmark of inflammation, appears to have an important role in hypoxic signaling and hypoxia inducible factor induction (Olson and van der Vliet 2011) and a recent review concluded that increased nitric oxide synthesis is associated with an improved response to hypoxia (Beall et al. 2012). Variations in the regulation of this inflammatory response might, therefore, explain the differing VOC profiles between AMS‐resistant and AMS‐susceptible individuals.

### Relevance for critical illness

Analysis of exhaled breath VOCs may have potential to be developed into an objective prognostic and diagnostic tool for not only AMS, but also other clinical conditions. If breath analysis can identify the poor responders to hypobaric hypoxia it may also have utility in identifying those maladapted to *normo*baric hypoxia e.g., in critical illness. Furthermore, if we could identify which volatiles dominate the differences between individuals then this may offer a mechanistic insight into the cellular processes involved and thereby help explain the pathogenesis of hypoxia (mal)adaptation.

## Conclusions

In this study we have demonstrated the feasibility of a method for exhaled VOC analysis in a remote high altitude environment using an e‐nose (Cyranose 320). Our results provide proof‐of‐concept for its use as an objective tool in the prediction and diagnosis of AMS. The development of such a tool could have major impacts on altitude medicine as well as other clinical areas. The ability to differentiate the breathprints of Sherpas from lowlanders supports the use of breath analysis to discriminate between different (patho)physiological responses to hypoxic conditions. It also suggests that analysis of exhaled breath may provide us with a route to better mechanistic understanding of hypoxic (mal)adaptation. Translational research to explore exhaled breath biomarkers in critical illness could help the development of improved, phenotype‐specific management strategies for our sickest patients.

## Conflict of Interest

JL, CK, JvdK, PB, EG‐K declare that they have no competing interests.

MG is Joint Editor‐in‐Chief, Extreme Physiology and Medicine; Associate Editor, Perioperative Medicine; Medical Advisory Board, Sphere Medical Ltd; Board and Research Council, National Institute of Academic Anaesthesia; National Specialty Lead, NIHR Anaesthesia, Perioperative Medicine and Pain Specialty Group; Council, Royal College of Anaesthetists; Board, Faculty of Intensive Care Medicine; Chair, National Adult Critical Care Data Group; International Advisory Board, American Society of Enhanced Recovery; Board, Enhanced Recovery after Surgery UK; Chair, Xtreme Everest Oxygen Research Consortium; Chair, Fit‐4‐Surgery Research Collaboration; recipient of unrestricted grant to institution – Sphere Medical Ltd; honorarium and travel support for lecture – Edwards Lifesciences; funds for travel and accommodation – Smiths Medical Ltd.

MM is founding Editor‐in‐Chief of Perioperative Medicine; Editorial Board, British Journal Anaesthesia; Editorial Board, Critical Care; Consultant, Edwards Lifesciences and Deltex Medical; Director, Medinspire Ltd; Director, Clinical Hydration Solutions Ltd; Director, Bloomsbury Innovation Group CiC; Director, Evidence‐based Perioperative Medicine CiC; International Advisory Board, American Society of Enhanced Recovery; Board member, Xtreme Everest Oxygen Research Consortium; Council, Royal College of Anaesthetists; Council Member and Chair, Board of the National Institute of Academic Anaesthesia; Smiths Medical Professor of Anaesthesia and Critical Care at University College London; recipient of funds for travel and accommodation from Edwards Lifesciences and Deltex Medical.

DM has received consultancy fees from Siemens Healthcare.
